# The Influence of Drugs upon Stomach Digestion

**Published:** 1886-11

**Authors:** 


### *THE INFLUENCE OF DRUGS UPON STOMACH DIGESTION.*

Klikowitch has performed a series of experiments to ascertain the effect of certain drugs upon the digestive powers of the gastric juice. In order to prepare gastric juice artificially, the author dissolves from five centigrams to a gram of pepsine in an officinal (F. P.) solution of chlorhydric acid, (10 cu. centimetres of acid in a litre of water.) From twenty to forty grams of albumen were added to 450 grams of the artificial gastric juice, and in this solution the preparation of the medicine to be tested was placed. The quantity of peptones was determined by means of the apparatus of Soleil-Wentzke and of Larant. The following is a summary of the results obtained:

1. *Alcohol.*—Peptonization is not influenced by this substance, unless the amount of alcohol in the digestive mixture exceeds five per cent. When ten per cent of alcohol is present, digestion is retarded, and digestion ceases when more than ten per cent. of alcohol is placed in the digestive mixture.

2. *Antipyrine.*—When twenty-five centigrams of this drug are added to two grams of the digestive mixture, the process of peptonization is not affected. A larger dose retards digestion, but not to a very marked extent.

3. *Bromide and Iodide of Potassium.*—In small doses, half a gram, no effect is produced. A dose of one or two grams slightly retards digestion. The iodides retard peptonization to a greater extent than the bromides.

4. *The Salts of Iron*—The salts of the organic acids do not affect digestion; reduced iron and the salts of the inorganic acids arrest peptonization in a remarkable manner.

5. *Calomel* diminishes peptonization, in doses of from three decigrams to a gram.

6. *Arseniate of Soda.*—No injurious effect when the dose is small (from five centigrams to a decigram)—contrary to what we would expect considering the bad reputation of the drug.

7. *Salicylate of Soda.*—Arrests peptonization in a very marked manner in doses of from twenty-five centigrams to five grams.

8. *Sulphate of Soda and Magnesia* even in small doses diminishes peptonization.

9. *Hydrate of Chloral*, in doses of less than one gram, does not influence stomach digestion. A larger dose (from one to five grams) will greatly retard digestion.

10. *Chloride of Sodium* will not affect digestion unless the dose exceeds one gram. In larger doses, peptonization is somewhat retarded.

11. *Chloride of Potassium.*—Same results.—*Bull. Gen. de Therapeutique.*

